# The One-Hundred-Year Anniversary of the Discovery of the Sunshine Vitamin D_3_: Historical, Personal Experience and Evidence-Based Perspectives

**DOI:** 10.3390/nu15030593

**Published:** 2023-01-23

**Authors:** Michael F. Holick

**Affiliations:** Department of Medicine, Boston University Chobanian & Avedisian School of Medicine, Boston, MA 02118, USA; mfholick@bu.edu; Tel.: +1-617-358-6139

**Keywords:** vitamin D, McCollum, sunlight, rickets, 25-hydroxyvitamin D, 1,25-dihydroxyvitamin D, food fortification, vitamin D receptor, vitamin D_2_, vitamin D_3_, COVID-19

## Abstract

The discovery of a fat-soluble nutrient that had antirachitic activity and no vitamin A activity by McCollum has had far reaching health benefits for children and adults. He named this nutrient vitamin D. The goal of this review and personal experiences is to give the reader a broad perspective almost from the beginning of time for how vitamin D evolved to became intimately involved in the evolution of land vertebrates. It was the deficiency of sunlight causing the devastating skeletal disease known as English disease and rickets that provided the first insight as to the relationship of sunlight and the cutaneous production of vitamin D_3_. The initial appreciation that vitamin D could be obtained from ultraviolet exposure of ergosterol in yeast to produce vitamin D_2_ resulted in the fortification of foods with vitamin D_2_ and the eradication of rickets. Vitamin D_3_ and vitamin D_2_ (represented as D) are equally effective in humans. They undergo sequential metabolism to produce the active form of vitamin D, 1,25-dihydroxyvitamin D. It is now also recognized that essentially every tissue and cell in the body not only has a vitamin D receptor but can produce 1,25-dihydroxyvitamin D. This could explain why vitamin D deficiency has now been related to many acute and chronic illnesses, including COVID-19.

## 1. Prequel

The sunshine vitamin D is likely to be the oldest hormone to be produced almost from the beginning of time when eukaryotes began to evolve in the oceans more than 1–1/2 billion years ago. The first evidence for this was the observation that a phytoplankton species *Emiliania huxlei*, which has existed for more than 750 million years unchanged in the Sargasso Sea, contained ergosterol (provitamin D_2_; 1 mcg/g dry weight), which was converted to previtamin D_2_ when exposed to simulated sunlight. Why these early eukaryotic organisms would contain such a large amount of ergosterol and produce vitamin D_2_ is unknown. It has been speculated that early photosynthetic eukaryotes that required sun exposure for their energy source were also at risk of being exposed to ultraviolet radiation (UV) that could damage their precious DNA, RNA and proteins. The UV absorption spectra for provitamin D_2_, previtamin D_2_ and vitamin D_2_ have high-extinction coefficients that completely overlap the UV-absorption spectra for these UV-sensitive macromolecules, thereby protecting them from solar ultraviolet radiation damage. It was also suggested that ergosterol in the plasma membrane not only served as an ideal sunscreen but also, upon exposure to ultraviolet radiation, was converted to previtamin D_2_. Previtamin D_2_, which is a planar structure embedded into the membrane, is thermally unstable and isomerizes into a non-planar vitamin D_2_ structure. This process would have caused a significant structural change in the bilipid layer of the plasma membrane, resulting in vitamin D_2_ being released into the environment. This process would have opened a tiny pore in the membrane that could have permitted the entrance of calcium and other cations into the cell. This could help explain why sunlight, vitamin D and calcium are so intimately related. When vertebrate life forms left the oceans for terra firma, they were leaving a calcium-rich environment into a poor calcium environment. It is possible that the association of the production of vitamin D_2_ in the plasma membrane of a phytoplankton, permitting the entrance of calcium into the cell, may have been taken advantage of in land vertebrates. During exposure to sunlight, land vertebrates would have produced vitamin D_3_ in their skin, which would have entered their circulation and increased the efficiency of calcium absorption. Although it is unknown when vitamin D_3_ was first metabolized and activated, it is likely that this occurred as marine vertebrates began to leave the oceans and explore and ultimately dominate terra firma. Most vertebrates require sunlight for their vitamin D requirement to maintain a healthy skeleton. Some species such as cats, other heavily furred vertebrates, and mole rats obtain their vitamin D from dietary sources. Fish-eating bats and vampire bats easily obtain an adequate amount of vitamin D from their diet, and recently, it was reported that insects, including mosquitoes exposed to sunlight, produce vitamin D_3_ and are excellent dietary sources for insect-eating bats [1,2,3,4].

## 2. The Discovery of the Sunshine Vitamin D

**Figure 1:**
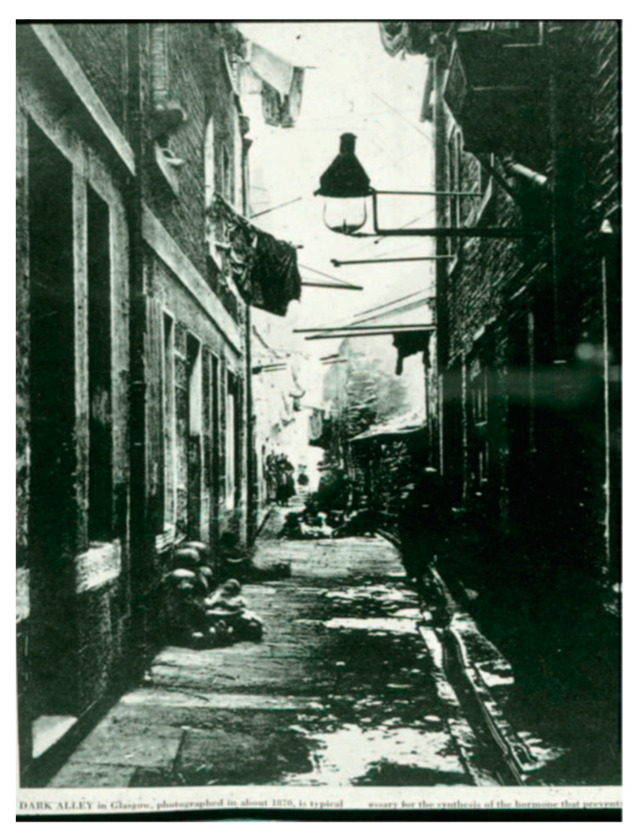
Photograph from Glasgow, Great Britain, in about 1870 showing that the buildings are built near each other. Ref. [5] Holick, copyright 1994. Reproduced with permission.

By the 16th century, there was a migration of the population into crowded and air-polluted cities (Figure 1). Simultaneous with this migration was the appearance of a devastating crippling bone-deforming disorder that affected most of the children.

**Figure 2:**
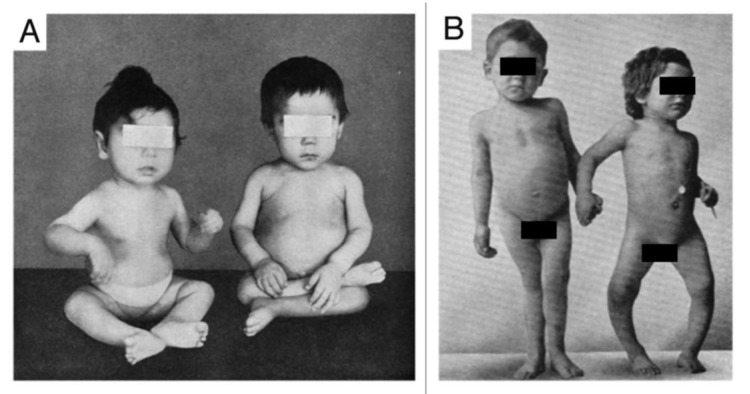
Skeletal deformities observed in rickets. (**A**) Photograph from the 1930s of a sister (left) and brother (right), aged 10 mo and 2.5 y, respectively, showing enlargement of the ends of the bones at the wrist, carpopedal spasm, and a typical “Taylorwise” posture of rickets. (**B**) The same brother and sister 4 y later, with classic knock knees (genu valgum) and bowed legs (genu varum), growth retardation, and other skeletal deformities. Ref. [7] Holick, copyright 2006. Reproduced with permission.

In 1650, Glisson, DeBoot and Whistler published their observations and concluded that this was a distinct disease that caused deformities of the skeleton, including enlargement of the joints of the long bones and rib cage, curvature of the spine and thighs, short stature, generalized muscle weakness and enlargement of the head (Figure 2). This disease, commonly known as rickets or English disease, continued to spread throughout European cities, and when the industrial revolution arrived in the 19th century, rickets became endemic in the northeastern United States. By the 20th century, it was estimated based on autopsy studies performed in Boston and Leiden that between 80–90% of children living in industrialized cities in Europe and the United States were suffering from this catastrophic bone-softening skeletal disorder. Besides causing bone deformities and growth retardation, vitamin D deficiency also gave rise to a flattened deformed pelvis with a small pelvic outlet in females of childbearing age. As a result, young women had a difficult time with child birthing, often experiencing serious complications including death of both the mom and newborn. In 1889, Murdock Cameron, a Scottish surgeon, began routinely performing C-sections to save both the mother and infant from a prolonged, difficult, and life-threatening childbirth [6,7,8].

The first association relating inadequate sunlight exposure as a cause for rickets was made by the Polish physician scientist Sniadecki in 1822 [9]. He connected the dots and realized that children living in industrialized Warsaw who were not exposed to direct sunlight were plagued with rickets, whereas children who lived in rural farm areas and were outdoors and exposed to sunlight had no evidence of this bone-deforming disease. This was followed by Palm who in 1890 recognized that the lack of sunlight was a common denominator that could be associated with the high incidence of rickets in children living in the inner cities in Great Britain when compared to children living in underdeveloped countries [10]. He encouraged systematic sunbathing as a means for preventing and curing rickets, as did Sniadecki [9].

It was common folklore in the 19th century in fishing communities on the coast of England to give their children cod liver oil as a way of preventing rickets. Bretonneau in 1827 treated a 15-month-old child with severe rickets with cod liver oil and noted the incredible speed at which the patient was cured. His student Trousseau further demonstrated that oils from aquatic mammals including seals and whales as well as oily fish including herring were also effective in treating rickets [7,8]. These observations suggested that rickets was caused by a nutritional deficiency. Finally, in 1918, Mellanby reported that he could produce rickets in beagles by placing them on an oatmeal diet and then reversing the disease by giving them cod liver oil [11]. This observation convinced the scientific community that rickets was caused by a nutritional deficiency and the hunt was on to determine what nutrient was deficient. It was originally concluded that vitamin A in cod liver oil was responsible for its antirachitic activity. However, Elmer McCollum, who had discovered vitamin B, was not convinced that the antirachitic factor was vitamin A. It was known, based on the observation by Hopkins, that vitamin A was very sensitive to heat and could easily be destroyed by heating and aerating it [8]. McCollum used this information and conducted a study where he aerated and heated cod liver oil and demonstrated that it no longer had vitamin A activity but was still capable of healing rickets. This eureka discovery resulted in McCollum naming this new nutrient vitamin D [12].

**Figure 3:**
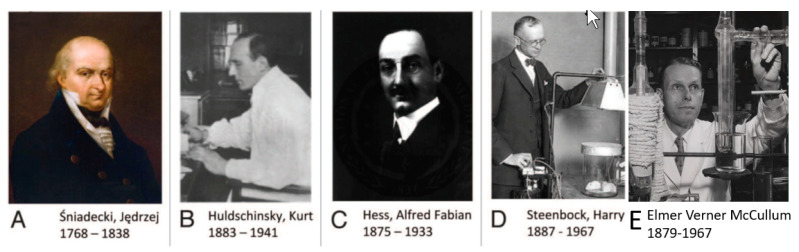
Photographs of researchers who made crucial contributions to vitamin D and rickets research. (**A**) Jędrzej Sniadecki, (**B**) Kurt Huldschinsky, (**C**) Alfred Hess, (**D**) Harry Steenbock, and (**E**) Elmer McCullum. Ref. [8] Holick, copyright 2013. Reproduced with permission.

**Figure 4:**
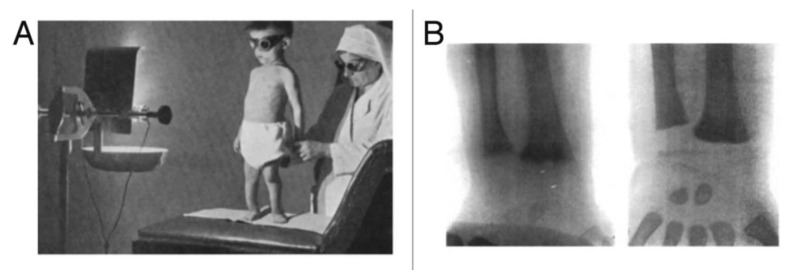
UV radiation therapy for rickets. (**A**) Photograph from the 1920s of a child with rickets being exposed to UV radiation. (**B**) Radiographs demonstrating florid rickets of the hand and wrist (**left**) and the same wrist and hand taken after treatment with 1 h UV radiation 2 times per week for 8 weeks. Note mineralization of the carpal bones and epiphyseal plates (**right**). Ref. [7] Holick, copyright 2006. Reproduced with permission.

At the same time, sunlight was beginning to be appreciated for its health-promoting properties. For his observations that direct sunlight exposure was effective in treating cutaneous tuberculosis and other skin disorders, Finsen received the Nobel Prize in 1903 [8]. In 1919, Huldschinski demonstrated that exposing children to radiation from a sun quartz lamp (mercury arc lamp) or carbon arc lamp resulted in marked increases in the mineralization of the long bones in the children’s X-rays. He evaluated a similar group of children not exposed to his lamps and reported no significant change in their X-rays. (Figure 3) He concluded that exposure to ultraviolet (UV) radiation was an infallible remedy against all forms of rickets in children. He also exposed one arm of some children to one of his lamps and demonstrated that the improvements in the mineralization in the X-rays of the children were identical in both arms. He concluded that the UV exposure to the skin was causing some type of photochemical reaction, producing a substance that entered the circulation and had a systemic effect on the skeleton [13,14]. These observations prompted Alfred Hess to expose seven rachitic children in New York City to varying periods of sunshine, and he reported marked improvement in rickets of each child as evidenced by calcification of their epiphyses (growth plates at the end of the long bones). He also concluded that children of color were at higher risk for developing rickets and required longer exposure times to sunlight to have the same effect that was observed in white children [15] (Figure 4).

**Figure 5:**
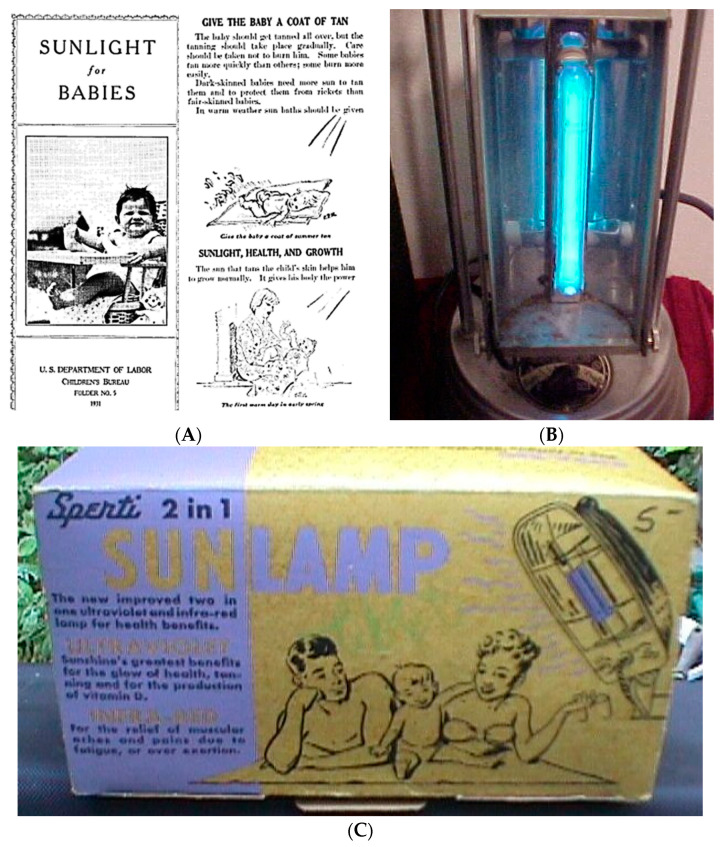
(**A**). Brochure from the US Department of Labor promoting sensible sun exposure for children in 1931. (**B**). Mercury arc lamp turned on for the use of preventing rickets in children in the 1930s. (**C**). The Sperti lamp available in the 1940s was used to expose children to ultraviolet radiation to promote bone health and to prevent rickets. Holick copyright 2023. Reproduced with permission.

**Figure 6:**
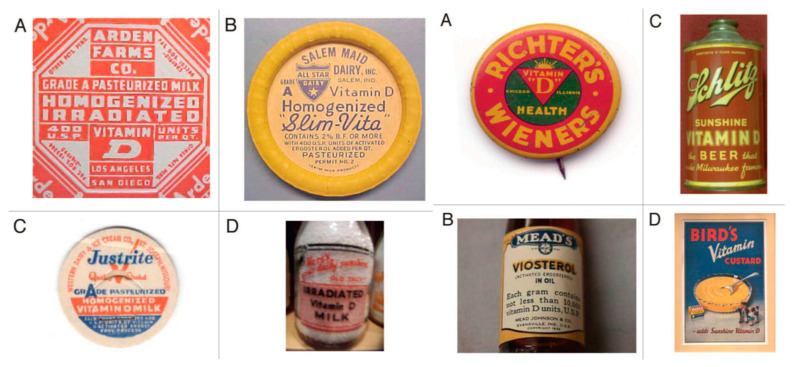
(**Left panel**). (**A**) Seal of a milk bottle that denoted that the milk was irradiated with UV radiation and contained vitamin D. (**B**) Cap of a milk bottle stating that activated ergosterol had been added to the milk. (**C**) Cap of milk bottle stating that the milk had been fortified with vitamin D. (**D**) Seal of a bottle of milk that denoted that the milk had been irradiated and contained vitamin D. Holick, copyright 2013. Reproduced with permission. (**Right panel**) (**A**) Seal denoting that this product was fortified with vitamin D. (**B**) Bottle of oil denoting that it contained irradiated ergosterol. (**C**) Beer can denoting that it was fortified with vitamin D. (**D**) Advertisement denoting that Bird’s custard contained vitamin D. Ref. [8] Holick, copyright 2013. Reproduced with permission.

These two disparate observations for the cure of rickets caused confusion. In 1921, Powers et al. fed a rachitic diet to rats and demonstrated that exposure to ultraviolet radiation was effective in preventing rickets, similar to dietary vitamin D [16]. However, the true appreciation for the health benefits of preventing rickets by exposing foods to UV radiation did not occur until 1924. Steenbock and Hess independently reported that exposure of foods, cotton seed and corn oil and especially the sterol fraction of foods with UV radiation produced antirachitic activity in rodents and chickens. Whereas Hess commented that “the question of the therapeutic value of this procedure is of secondary importance”, Steenbock patented the process for the UV irradiation of foods and other substances as a means of increasing their antirachitic properties. These included yeasts, cereals, grain, oils, fats, butter and milk. The dairy industry appreciated the importance of providing milk to children with antirachitic activity and initiated the addition of ergosterol, obtained from yeast, to milk, followed by UV irradiation. Later, UV-irradiated ergosterol was added to milk to provide antirachitic activity [17,18]. By the early 1930s, essentially all milk in the United States and most European countries including Great Britain encouraged the fortification of milk with vitamin D_2_. At the same time, the United States Department of Labor sent out a brochure encouraging parents to give their children a coat of tan from sun exposure to prevent rickets and to improve bone health (Figure 5). Within a few years, these interventions resulted in the essential eradication of rickets in the industrialized countries. Vitamin D fortification was so popular worldwide that beer, soda pop, hot dogs, custard and even soap and shaving cream were fortified with vitamin D (Figure 6). Parents were also able to purchase a mercury arc lamp from their local pharmacy and expose their children at home to ultraviolet radiation to prevent rickets (Figure 5).

The vitamin D craze abruptly came to a halt when, in the early 1950s, an outbreak of infants with elfin facies, mild mental retardation, cardiac problems, and hypercalcemia was reported. The Royal College of physicians initiated an investigation and concluded that this outbreak in children was due to ingestion of excessive amounts of vitamin D in milk. This was based on a published observation that pregnant rats that received high doses of vitamin D delivered pups with some of the same clinical presentations including altered facial appearance, supravalvular aortic stenosis and hypercalcemia. Since it was difficult to monitor the content of vitamin D in milk, as a precautionary measure and based on the recommendation from the Royal College of Physicians, legislation quickly followed banning the fortification of any food or personal use product with vitamin D [19,20]. This ban quickly spread across Europe, Asia, India, and South America. Most of these countries still ban the fortification of foods with vitamin D with a few exceptions. Finland and Sweden now permit milk, margarine, and some cereals to be fortified with vitamin D. India recently introduced in 2017 a vitamin D_2_ fortification program for milk and cooking oil. In retrospect, it is more likely that the children in Great Britain were suffering from the rare genetic disorder, Williams syndrome, which is associated with elfin facies, supravalvular aortic stenosis, mild mental retardation, and a hypersensitivity to vitamin D that causes hypercalcemia [21]. Unfortunately, this ban on vitamin D fortification persists in many countries throughout the world. It is responsible for the increased risk of rickets in children and vitamin D deficiency and insufficiency in children and adults.

## 3. Structural Identification of Vitamin D_2_ and Vitamin D_3_

When the oily sterol extract from yeast, containing ergosterol, was exposed to UV radiation, it was assumed that this resulted in the production of the antirachitic factor, vitamin D. However, it was quickly realized that there were other photoproducts present in the irradiated oil, and therefore, the UV-exposed sterol oil was called visterol. (Figure 6, right panel B). When animal oily sterols that mainly contained cholesterol were exposed to UV radiation, it resulted in the oils processing antirachitic properties. It was presumed that there was the contaminant, ergosterol, in the cholesterol oil that was transformed after UV irradiation into vitamin D. In 1932, Windaus in Germany and Bourdillon in England isolated pure crystalline vitamin D from irradiated ergosterol. Windaus and his colleagues named the compound vitamin D_2_, whereas the British investigators called their crystalline compound ergocalciferol [22,23]. Originally irradiated ergosterol was given the name vitamin D_1_. Once it was realized that the irradiated ergosterol contained vitamin D and lumisterol, the term was no longer used.

**Figure 7:**
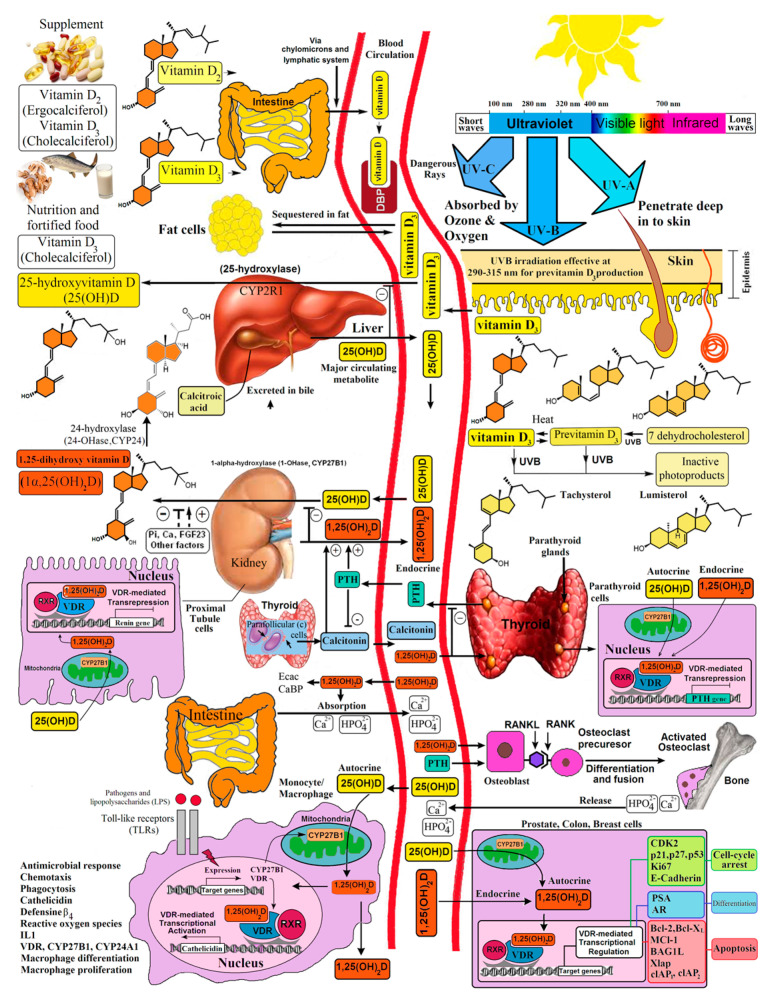
Schematic representation of the synthesis and metabolism of vitamin D for skeletal and extra-skeletal function. During exposure to sunlight, 7-dehydrocholesterol in the skin is converted to previtamin D_3_. Previtamin D_3_ immediately converts by a heat-dependent process to vitamin D_3_. Excessive exposure to sunlight degrades previtamin D_3_ and vitamin D_3_ into inactive photoproducts. Vitamin D_2_ and vitamin D_3_ from dietary sources are incorporated into chylomicrons and are transported by the lymphatic system into the venous circulation. Vitamin D (D represents D_2_ or D_3_) made in the skin or ingested in the diet can be stored in and then released from fat cells. Vitamin D in the circulation is bound to the vitamin D-binding protein (DBP), which transports it to the liver, where vitamin D is converted by the vitamin D-25-hydroxylase to 25-hydroxyvitamin D [25(OH)D]. This is the major circulating form of vitamin D that is used by clinicians to measure vitamin D status (although most reference laboratories report the normal range to be 20–100 ng/mL, the preferred healthful range is 30–60 ng/mL). It is biologically inactive and must be converted in the kidneys by the 25-hydroxyvitamin D-1a-hydroxylase (1-OHase) to its biologically active form 1,25-dihydroxyvitamin D [1,25(OH)_2_D]. 1,25(OH)_2_D_3_ is then taken up by target cells and targeted to intracellular D-binding proteins (IDBP) to mitochondrial 24-hydroxylase or to the vitamin D receptor (VDR). The 1,25(OH)_2_D_3_-VDR complex heterodimerizes with the retinoic acid receptor (RXR) and binds to specific sequences in the promoter regions of the target gene. The DNA-bound heterodimer attracts components of the RNA polymerase II complex and nuclear transcription regulators. Serum phosphorus, calcium fibroblast growth factors (FGF-23), and other factors can either increase or decrease the renal production of 1,25(OH)2D. 1,25(OH)_2_D feedback regulates its own synthesis and decreases the synthesis and secretion of parathyroid hormone (PTH) in the parathyroid glands. 1,25(OH)_2_D increases the expression of the 25-hydroxyvitamin D-24-hydroxylase (24-OHase) to catabolize 1,25(OH)_2_D to the water-soluble, biologically inactive calcitroic acid, which is excreted in the bile. 1,25(OH)_2_D enhances intestinal calcium absorption in the small intestine by stimulating the expression of the epithelial calcium channel (ECaC) and calbindin 9K (calcium-binding protein, CaBP). 1,25(OH)_2_D is recognized by its receptor in osteoblasts, causing an increase in the expression of the receptor activator of the NF-kB ligand (RANKL). Its receptor RANK on the preosteoclast binds RANKL, which induces the preosteoclast to become a mature osteoclast. The mature osteoclast removes calcium and phosphorus from the bone to maintain blood calcium and phosphorus levels. Adequate calcium and phosphorus levels promote mineralization of the skeleton. Autocrine/paracrine metabolism of 25(OH)D occurs when a macrophage or monocyte is stimulated through its toll-like receptor 2/1 (TLR2/1) by an infectious agent such as Mycobacterium tuberculosis or its lipopolysaccharide the signal upregulates the expression of VDR and 1-OHase. A 25(OH)D level of 30 ng/mL or higher provides adequate substrate levels for 1-OHase to convert 25(OH)D to 1,25(OH)_2_D in mitochondria. 1,25(OH)_2_D travels to the nucleus, where it increases the expression of cathelicidin, a peptide capable of promoting innate immunity and inducing the destruction of infectious agents such as M. tuberculosis. It is also likely that the 1,25(OH)_2_D produced in monocytes or macrophages is released to act locally on activated T lymphocytes, which regulate cytokine synthesis, and activated B lymphocytes, which regulate immunoglobulin synthesis. When the 25(OH)D level is approximately 30 ng/mL, the risk of many common cancers is reduced. It is believed that the local production of 1,25(OH)_2_D in the breast, colon, prostate, and other tissues regulates a variety of genes that control proliferation, including p21. Ref. [26] Holick, copyright 2013. Reproduced with permission.

It was originally believed that vertebrate animals such as pigs and humans produced vitamin D_2_ in their skin when exposed to sunlight. Waddell, in 1934, found that irradiated ergosterol was less effective as an antirachitic factor in chickens when compared to the same amount given to rachitic rodents. He concluded that there was a contaminant in cholesterol, and it was not ergosterol, that was responsible for the antirachitic activity in cholesterol oil exposed to UV radiation [24]. This conundrum was finally solved when Windaus and colleagues synthesized 7-dehydrocholesterol, and after its irradiation, the isolated vitamin D had a high antirachitic activity in chickens, whereas vitamin D_2_ had only 1–3% the activity in chickens that it had in rats. Further investigations revealed that the vitamin D isolated from halibut and blue fin tuna oil was identical to the vitamin D generated from 7-dehydrocholesterol. Windaus named his vitamin D as vitamin D_3_. He received the Nobel Prize in 1928 for his work on the constitution of sterols, including the identification of vitamins associated with them. Schenck, in 1937, finally determined that Windaus’s vitamin D in his irradiated sterols was vitamin D_3_ based on its successful crystallization [25]. The structures for vitamin D_2_ and vitamin D_3_ can be found in Figure 7.

## 4. Understanding How Vitamin D Works to Promote Bone Health

By 1940, it was acknowledged that rickets could be prevented and cured in two ways: by irradiation of the skin to solar or artificial UV radiation or by the ingestion of vitamin D (D represents D_2_ or D_3_). It was recognized that rachitic children had low blood concentrations of phosphate and calcium and that treatment with vitamin D corrected them to normal. It was unclear however how vitamin D affected this. In 1923, Orr et al. observed decreased fecal excretion of calcium in rats exposed to UV radiation. They concluded that this was likely due to vitamin D increasing intestinal calcium absorption [27]. This was confirmed in 1953 by Nicolaysen et al. who established that the important function of vitamin D was for stimulating intestinal calcium absorption [28]. It was also generally believed that vitamin D was playing a fundamental role in bone mineralization. However, studies by Carlsson demonstrated that vitamin D, rather than stimulating the direct deposition of calcium into bone, stimulated the mobilization of calcium from the skeleton [29]. We now recognized that the fundamental function of vitamin D is to maintain the serum calcium and phosphate in the normal range for maintaining most metabolic functions, including neuromuscular and cardiac activity [7,30]. Vitamin D also maintains an adequate circulating and extracellular calcium–phosphate product, resulting in the passive deposition of calcium hydroxyapatite, i.e., mineral into the skeleton [7,30]. There is also evidence that vitamin D enhances intestinal phosphate absorption, which is also critically important for energy and muscle function through the action of ATP and is a major component of calcium hydroxyapatite in the skeleton (Figure 7) [30].

## 5. The Saga for the Quest to Identify the Active Form of Vitamin D_3_

**Figure 8:**
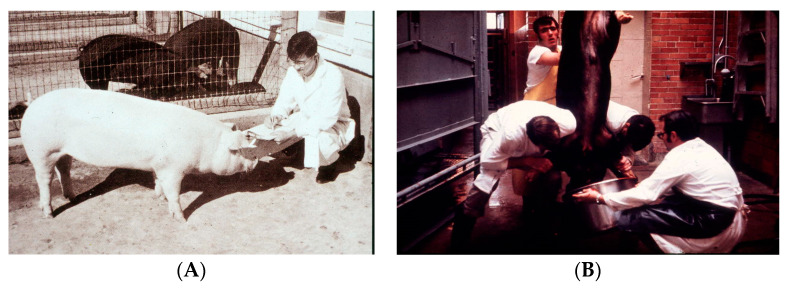
(**A**). This hog was treated with 6.25 mg vitamin D_2_ by Dr. Suda; (**B**) the blood was then collected. After purification of the blood lipid extract, the vitamin D_2_ metabolite was identified as 25-hydroxyvitamin D_2_. Holick copyright 2023. Reproduced with permission.

In the early 1950s, using radiolabeled calcium, it was determined that when a vitamin-D-deficient rat or chicken received vitamin D, intestinal calcium absorption began to increase, with maximum absorption occurring at around 24 h. Kodicek and coworkers reasoned that to understand the mode and sites of action of vitamin D, investigations were needed to understand its storage, distribution and metabolism. They produced ^14^C-beled vitamin D_2_ with a low specific activity of 0.46 mCi/mmol and administered pharmacologic doses to rats. They concluded, based on determination of radioactivity in the tissues and excreta by reverse chromatography, that vitamin D_2_ was likely the biologically active form of the vitamin [31]. In the early 1960s, Norman and DeLuca prepared randomly labeled ^3^H-vitamin D_3_ and ^3^H-vitamin D_2_ with much higher specific activities of 7.3 and 3.25 mCi/mmol, respectively, and gave them to vitamin-D-deficient rats. The chromatography results from their study revealed that there was accumulation of radioactivity mainly in the liver and to a lesser degree in the ileum and kidneys and much less in the adrenal glands. This resulted in the investigation of the metabolism of tritiated vitamin D_3_ in rat kidney and intestinal mucosa. At least three lipid-soluble radioactive compounds other than ^3^H-vitamin D_3_ were detected, and all showed partial vitamin D activity [32]. The DeLuca group then made [1, 2-^3^H] vitamin D_3_ with very high specific activity and found by silicic acid chromatography a polar metabolite labeled as metabolite IV circulating in rats. They found that this metabolite had at least the same antirachitic potency as vitamin D_3_. Furthermore, they discovered that the amount of this metabolite continued to increase with increasing doses of vitamin D_3_. Utilizing this fact, Blunt et al. gave four hogs 6.25 mg of vitamin D_3_ daily for 26 days and then collected their blood. They separately gave a hog [1, 2-^3^H] vitamin D_3_ and collected its blood. Organic extractions were made of both blood pools and were then combined. The lipid extract underwent a series of straight-phase and reverse-phase chromatographies, resulting in the recovery of 1.2 mg of metabolite labeled as peak IV. The vitamin D_3_ metabolite was identified as 25-hydroxyvitamin D_3_ [33]. Suda et al. performed a similar procedure, only this time using pharmacologic amounts of vitamin D_2_ and tritiated vitamin D_2_ and identified the polar metabolite as 25-hydroxyvitamin D_2_ (Figure 8) [34]. These observations in pigs were interesting, but it was unknown whether this metabolism also occurred in humans.

## 6. A Personal Experience and Perspective for the Identification of the Major Circulating Form of Vitamin D_3_ in Human Blood

Clearly, a different strategy would be needed to determine if 25-hydroxylated vitamin D was present in human circulation. I arrived at the University of Wisconsin Madison in 1968 in the Department of Medical Microbiology. This was a time when there was great excitement about the discovery of DNA and RNA. Molecular biology was in its infancy, and my goal was to achieve a Ph.D. degree by investigating the molecular biology of microorganisms. However, no one in the department had any interest in this area, and I, after completing the year, went to the Biochemistry Department and explained to them that I had been accepted into the University’s graduate program and that they should accept me into the graduate program in the Biochemistry Department. Much to my amazement, I was accepted. I quickly realized, however, that I had a big problem to overcome. I was considered to have completed my first year of graduate work, and to proceed into the second year, I needed to take the biochemistry preliminary exam. Although I had a chemistry degree with a special interest in organic chemistry and had biochemistry laboratory experience, I had never taken a biochemistry course. As a result, I barely passed the exam. I wanted to find a research mentor who had a special interest in molecular biology. However, the professors, after seeing my poor performance on the preliminary exam, informed me that there were no openings but that I should see Professor DeLuca, who was making important observations about vitamin D. I explained to various professors that I had no interest in vitamin D since I considered it a boring subject. I was informed that this was my only option. When I met Dr. DeLuca in June 1969, he was pleased that I had significant biochemistry research experience and a publication while I was in the chemistry program at Seton Hall University. However, after seeing my preliminary exam results, he informed me that I needed to go into a softer science program such as physiology or pharmacology and that biochemistry would be too difficult for me. I reassured him that it was my goal to receive a Ph.D. degree in biochemistry and that I would do whatever was necessary to accomplish this. He said that he did not want to discourage me but to be realistic, I needed to first receive a master’s degree that would likely take 2 years followed by being quizzed by the biochemistry faculty to determine if I could proceed into the Ph.D. program. He said that the Ph.D. program would take at least 4–5 years. Thus, he said it could take up to 7 years before I would receive a Ph.D. degree. I accepted the challenge, and he accepted me into his research program in July 1969. As the newest graduate student, I was given a desk against the wall where the entrance was located with no laboratory space. In July of 1969, Dr. DeLuca received a phone call from Dr. Avioli, at Washington University in St. Louis, informing him that he has been treating hypoparathyroid patients with pharmacologic doses of vitamin D_3_ (1.5 mg daily) and had been collecting their heparinized blood plasma. He asked Hector if he would like the blood plasma to see if his laboratory could identify 25-hydroxyvitamin D_3_ in the blood plasma. Since all his graduate students and postdoctoral fellows had ongoing research projects and I had none, I was informed by Dr. DeLuca that this would be my project. He instructed me that all I needed to do was to follow the exact chromatography procedures that were successfully used to identify 25-hydroxyvitamin D_2_ and 25-hydroxyvitamin D_3_ in hog blood. With great enthusiasm, I began by obtaining ^3^H-tracer from the blood of vitamin-D-deficient rats that were injected with [1, 2-^3^H] vitamin D_3_. I performed a lipid extraction on 600 mL of human blood plasma and rat serum and combined the lipid extracts. I proceeded to follow the exact chromatography methods used to successfully obtain purified 25-hydroxyvitamin D from hog blood. I conducted all the chromatographies that were previously reported. After exhausting all chromatographic procedures known at the time, I realized at the end of November 1969 that there was a lipid contaminant in human blood that was not present in hog blood and that the contaminant could not be separated from the vitamin D metabolite for its identification. This was Thanksgiving weekend. Since I could not afford to travel home to New Jersey to be with my family, I went to the laboratory. I unlocked the door early in the morning and walked in distressed because of my predicament of failing to purify the vitamin D metabolite. As I was walking to my desk, I noticed on one of the shelves on the research bench that was next to my desk a bottle containing Sephadex LH-20. I remembered from my college days that Sephadex, which is a glucose polymer, can separate proteins based on size. I reasoned that maybe I could use Sephadex LH-20 to separate the vitamin D metabolite from other contaminants based on size. I made a slurry of Sephadex LH-20 with methanol and poured the slurry into a column. After the Sephadex LH-20 settled, I placed my human plasma blood lipid extract, which had undergone multiple chromatographies, on the column and collected fractions. The vitamin D metabolite peak was identified by determining the location of the tritiated peak IV. The peak was recovered and the metabolite was now pure. I recovered 25 mcg that was identified as 25-hydroxyvitamin D_3_ (25(OH)D_3_). This metabolite is the major circulating form of vitamin D_3_ that is clinically measured to determine a person’s vitamin D status. What I did not realize at the time was that I had developed a new chromatography method known as liquid gel chromatography that became the standard chromatography technique in most vitamin D laboratories and was instrumental for purifying the active form of vitamin D_3_ [35,36]. I had completed my master’s research in 3 months, and for these discoveries, I was awarded a master’s degree in January 1970.

## 7. The Hunt to Identity the Active Form of Vitamin D_3_

It was found that 25(OH)D_3_ was biologically more potent than vitamin D_3_, and it was concluded that it was the active form [33]. However, it soon became evident that ^3^H-25(OH)D_3_ was rapidly metabolized to a more polar metabolite that quickly appeared in the intestine. Haussler et al. [37] reported a more polar metabolite of 25(OH)D_3_ in nuclear fractions of the intestine in chickens that received ^3^H-vitamin D_3_. At the same time, Lawson et.al. [38] reported that during the formation of this more polar metabolite, the 1^−3^H on their [4-^14^C,1α-^3^H] vitamin D_3_ was lost, suggesting that this polar metabolite had some alteration on carbon 1. The realization that this more polar metabolite not only had all the biologic actions that vitamin D_3_ and 25(OH)D_3_ possessed but that it acted more quickly in stimulating intestinal calcium absorption. It became clear that 25(OH)D_3_ was not the biologically active form, but rather, it was a more polar metabolite that needed to be identified.

**Figure 9:**
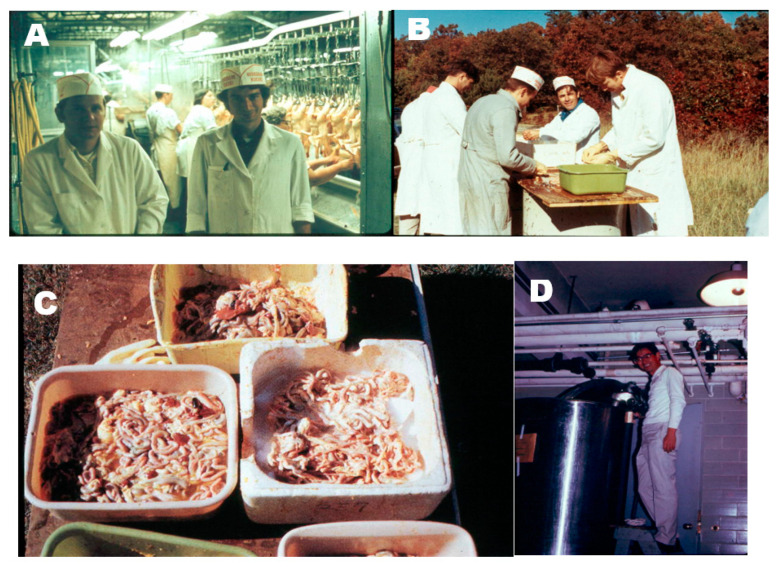
(**A**) The first attempt. Dr. Robert Cousins on the left and me collecting intestines from the trough behind us at the BrakeBush facility. (**B**) The second collection of intestines with several members of the DeLuca laboratory helping out. From left to right is Dr. Ian Boyle, Dr. Chuck Frolich, Dr. Jack Omdahl and Dr. Hector DeLuca. (**C**) Cleaned and processed intestines placed on dry ice. (**D**) Dr. Tatsuo Suda inspecting the huge stainless steel fermentation tank that contained approximately 400 pounds of mashed intestines and several hundred gallons of chloroform and methanol for the lipid extraction. Holick copyright 2023. Reproduced with permission.

This realization initiated a frenzy of activity in the Kodicek, Norman and DeLuca laboratories. Various strategies were undertaken, the first being in the DeLuca lab where they gave hogs pharmacologic doses of vitamin D_3_ and then collected their intestines. Dr. Robert Cousins, who was a postdoctoral fellow in DeLuca’s laboratory, was in charge. It was quickly realized that it made little sense to be giving pharmacologic doses of vitamin D_3_ and expecting marked increases in the concentrations of the active form in the intestine. As a result, in the summer of 1970, it was decided that what was needed was many intestines from chickens that were receiving presumably physiologic amounts of vitamin D_3_ in their diet. Dr. Cousins, Dr. Suda and I traveled an hour north in the department of biochemistry’s pickup truck to the BrakeBush facility where they processed 20,000 chickens per day (Figure 9A). We collected approximately 400 pounds of chicken intestines and returned to the University of Wisconsin with the intestines. We quickly realized that the intestines had begun to putrefy since we had not brought ice with us, and it was a hot steamy day. It was decided that the entire laboratory would go back to the BrakeBush facility to collect, clean, and process approximately 400 pounds of chicken intestines and freeze them immediately on dry ice (Figure 9B,C). The realization quickly set in upon arriving back at the University that we had no ability to process such a large amount of intestinal material. The biochemistry department had large fermentation stainless steel containers in the basement. Dr. Cousins, Dr. Suda and I began to process the intestines in a sausage meat grinder. The mashed intestines were then added to a large fermentation tank (Figure 9D). Several hundred gallons of chloroform, methanol and water were added and stirred. An additional amount of chloroform was then added, which caused a separation of the organic phase and which contained the lipids that were present in the intestines, from the water phase, which contained the water-soluble compounds. The several hundred gallons of the chloroform phase was taken to dryness and then chromatographed on a huge Shepadex LH-20 column that I designed. It became obvious to me that it was going to be futile to chromatograph and purify the minute quantity of the active form of vitamin D_3_ that was present in several hundred grams of intestinal lipid extract. 

**Figure 10:**
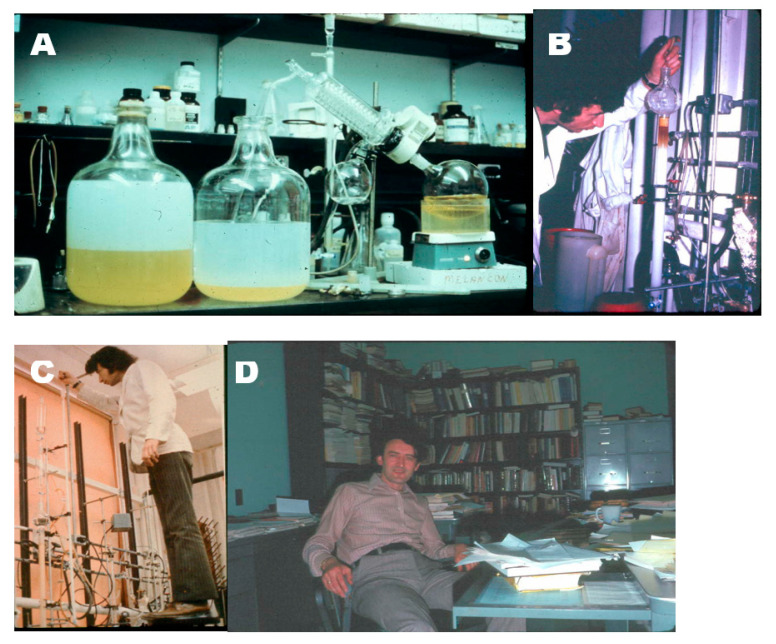
(**A**) Lipid extraction of 1450 chicken intestines. The yellow chloroform organic phase that contained the active metabolite was being dried down under vacuum in preparation for its first chromatography on a Shephadex LH-20 column (**B**) The dried down lipid extract from the intestines was first chromatographed on a Shephadex LH-20 column and eluted with a mixture of 65% chloroform in hexane. (**C**) In one of the final chromatographies, the semi-purified active vitamin D_3_ metabolite was dissolved in methanol and chromatographed on a Shephadex LH-20 column prepared in methanol. (**D**) Dr. Heinrich Schnoes in his office. Holick copyright 2023. Reproduced with permission.

**Figure 11:**
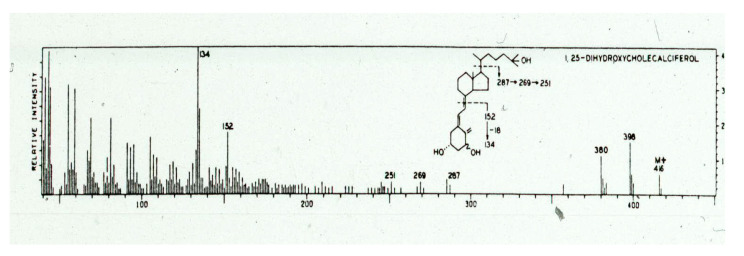
The mass spectrum of the purified metabolite demonstrated a molecular ion of 416 *m*/*e* consistent with an additional hydroxyl group on the metabolite. The fragments of 152 and 134 revealed that the hydroxyl group was somewhere in the A ring, most likely on carbon 1. Several chemical reactions were performed on nanogram quantities of the pure metabolite that provided evidence that the extra hydroxyl group was on carbon 1. Holick copyright 2023. Reproduced with permission.

Thus, while this process was underway, I went to Dr. DeLuca and suggested to him that we know based on multiple studies that there was approximately 1 ng of the active metabolite in 1 g of intestine. I suggested to him that a better way of isolating this intestinal metabolite was to give vitamin-D-deficient chickens intravenously 100 IUs of vitamin D_3_ that contained [1, 2-^3^H] vitamin D_3_. Based on my calculation, I explained to him that I would need 1500 chicken intestines to obtain approximately 10 mcg of the active metabolite that could then be extracted, chromatographed, and ultimately purified for identification. He gave me the green light. I asked Dr. Jack Omdahl, who was a postdoctoral fellow in Dr. Deluca’s laboratory, with extensive experience in working with vitamin-D-deficient chickens, for his assistance. Due to the capacity of the department of agriculture to house chickens, I was able to only order 500 chicks. I placed them on a vitamin-D-deficient diet that we produced in the biochemistry department. After 6 weeks on the vitamin-D-deficient diet, Jack gave an oral dose of 100 IUs (2.5 mcg) of vitamin D_3_ containing trace amounts of [1, 2-^3^H] vitamin D_3_ in vegetable oil to all 500 chickens. The chickens were sacrificed 24 h later, and Jack and I removed and cleaned the small intestines and placed them in a freezer. At the same time, another 500 chickens were ordered and placed on the vitamin-D-deficient diet and underwent the same procedures. By November 1970, I had 1000 intestines stored in the freezer and was waiting for my last 450 chickens that were scheduled to be sacrificed in early December. However, at the end of November, late at night, we heard a news flash on BBC radio stating that the Kodicek laboratory had made a major discovery related to vitamin D. We did not know what that discovery was. Dr. Deluca became extremely concerned that this meant that the Kodicek group had successfully identified the active form of vitamin D. I was asked to proceed immediately to process the 1000 chicken intestines I had in the freezer, with the intent of identifying the active form of vitamin D_3_ as quickly as possible. My response was that I needed 1500 chicken intestines to be successful and that I did not want to proceed. Challenging my mentor was not easy but I initially stood my ground. I then relented, reiterating my concern that I would not be successful but that I would do as directed and begin the processing of the thousand chicken intestines the next morning. As I was beginning to collect the frozen intestines, early the next morning, Dr. DeLuca appeared and informed me that I had been right in the past with my research activities and that he would permit me to wait until I collected the additional 500 intestines. By the middle of December 1970, I had completed the lipid extraction of all 1450 chicken intestines. Based on the known specific activity of the [1, 2-^3^H] vitamin D_3_ that was given to the chickens, I estimated that I had approximately 11 mcg of the active metabolite in 22 g of oily lipid. I immediately began multiple chromatographies on my Sephadex columns (Figure 10A,B). It was now coming close to Christmas, and I realized that we were in stiff competition with the Kodicek and Norman laboratories. I flew home to New Jersey on Christmas Eve and wished my parents a Merry Christmas and flew back to Madison Wisconsin on Christmas day to continue to purify the active vitamin D_3_ metabolite that was called peak V. When I returned, I learned that the Kodicek group published its newsworthy finding in the journal Nature. At this time, it was assumed that because this active metabolite appeared so quickly in the intestine that it was the intestine that was producing it. Therefore, it was obvious that to generate the active form of vitamin D_3_ in sufficient quantities for its identification, all you would need to do is incubate intestinal homogenates with 25(OH)D_3_. After numerous attempts by several laboratories including the DeLuca laboratory, incubation of intestinal homogenates with [^3^H]-25(OH)D_3_ did not yield any of the desired polar metabolite. The Kodicek group not only made homogenates of chicken intestines, they made homogenates of many of the other organs. They then incubated each of the homogenates with [4-^14^C,1-^3^H] 25(OH)D_3_. What they observed was truly remarkable. They found that only kidney homogenates could produce the active metabolite [39]. For me, this revelation was very disconcerting, since it meant that an unlimited amount of active vitamin D_3_ could be easily produced by incubating kidney homogenates with 25(OH)D_3_. It was becoming increasingly more likely that the Kodicek group was close to purifying the active metabolite for its identification. By early January 1971, I realized after 17 chromatographies, I had approximately 8 mcg of active metabolite that was associated with a large amount, that I estimated to be about 2000 mcg, of lipid contamination (Figure 10C). There were no more chromatographies that I could do that would make any difference, and I concluded that I had failed. However, within a few hours, I reasoned that we knew that the active form of vitamin D had a secondary hydroxyl group on carbon 3 and a tertiary hydroxyl group on carbon 25. It was presumed that there was an additional secondary hydroxyl group on carbon 1 based on the observation by the Kodicek group and that this metabolite had lost its 1α−^3^H on carbon 1. I considered that it was unlikely that the lipid contaminants in my preparation had the same number and type of hydroxyl groups. I reasoned that if I trimethylsilylated the active metabolite, and then selectively, by acid hydrolysis, removed the trimetylsilyl ether groups from the secondary hydroxyl groups, this would result in the 25-hydroxyl being protected with a trimethylsilyl ether. This modification would decrease the polarity of the vitamin D metabolite, thereby altering its chromatographic properties. I proceeded with this strategy and chromatographed the dihydroxyated active form on a Sephadex LH-20 column and recovered it in pure form. The purified monosilyl ether derivative was then hydrolyzed, and after purification, 2 micrograms of pure metabolite was recovered. The ultraviolet absorption spectrum showed a lambda max 265 nm and a lambda min 228 nm that was identical to the absorption spectrum for the 5,6-triene system for vitamin D_3_. A small amount was immediately placed in the mass spectrometer, and it revealed a mass spectrum with a molecular ion of 416 *m*/*e.* This was consistent with an additional hydroxyl group being present in the metabolite. The fragmentation pattern was consistent with the metabolite having the additional hydroxyl group in the A ring (Figure 11). Further chemistry on nanogram quantities of the purified active form of vitamin D_3_, followed by the skillful mass spectroscopy by Dr. Heinrich Schnoes (Figure 10D), finally revealed its structure as 1,25-dihydroxyvitamin D_3_ (1,25(OH)_2_D_3_) (Figure 7 and Figure 11) [40].

During that same time, the Kodicek group isolated 60 mcg of the metabolite with approximately 30% purity, using Sephadex LH-20 chromatography, from chicken kidney homogenates. They reported that the isolated metabolite had a UV absorption lambda maximum of 269 nm and a lambda minimum at 232 nm. The mass spectrum showed a molecular weight of 416, identical to what we had observed. Because they only had 30% purity, the only way they were able to conclude that the additional hydroxyl group was on carbon 1 was based on their observation that the 1-^3^H on their [4-^14^C,1α-^3^H] vitamin D_3_ was lost while it was being metabolized. They concluded that their metabolite was also 1,25-dihydroxyvitamin D_3_ [41]. The Norman group also used the Kodicek kidney homogenate strategy and confirmed several months later that the structure of the active form of vitamin D was 1,25-dihydroxyvitamin D_3_. [42] These three observations were submitted for publication on 12 February to PNAS, 19 February to Nature and 10 May 1971 to Science, respectively [40,41,42]. We had won the race in just one week. For my research accomplishments, I received my Ph.D. Degree in May 1971, a little less than 2 years after I joined the DeLuca laboratory.

The final evidence that the one hydroxyl group was on C-1 was confirmed by the 21- step synthesis of 1,25-dihydroxyvitamin D_3_ by my roommate Eric Semmler and me [43]. We demonstrated that the synthetic metabolite migrated identically with the biologically produced active metabolite and had the same biologic activity on intestinal calcium absorption and bone calcium mobilization [43]. This was further confirmed several years later when my group made 1β, 25-dihydroxyvitamin D_3_ and demonstrated that it was biologically inactive [44]. Soon after these discoveries, Dr. Glennville Jones, while a postdoctoral fellow in Dr. DeLuca’s laboratory, began using the newly introduced high-performance liquid chromatography and identified 1,25-dihydroxyvitamin D_2_ as the biologically active form of vitamin D_2_. He and other investigators went on to identify numerous other vitamin D metabolites [45]. However, to date, it remains that 1α,25-dihydroxyvitamin D_3_ is the biologically active form of vitamin D_3_. 

## 8. Clinical Uses for 1α,25-Dihydroxyvitamin D_3_ and 1α-Hydroxyvitamin D_3_

Once 1,25-dihydroxyvitamin D_3_ was identified, there was a great interest in producing it chemically. It was decided that it would be worthwhile to see if the 1α-hydroxyl group could be introduced into the less expensive starting material, cholesterol. The synthesis of 1α-hydroxyvitamin D_3_ was accomplished. The same methodology was then used to successfully produce 1,25-dihydroxyvitamin D_3_ [46]. 1α-hydroxyvitamin D_3_ was shown to be biologically active after it was metabolized in the liver on carbon 25 to form 1,25-dihydroxyvitamin D_3_ [47]. 

Several milligrams of both compounds were produced. They were immediately sent out to clinicians around the world who found that these active vitamin D compounds were extremely effective in treating metabolic calcium and bone diseases in children and adults with kidney failure [48,49]. It was known that kidney-failure patients had a resistance to the biologic actions of vitamin D that was difficult to understand. The revelation that the kidneys were responsible for metabolizing 25(OH)D_3_ to 1,25-dihydroxyvitamin D_3_ revealed the reason why. It was also observed that a rare form of hereditary rickets, known as vitamin-D-dependent rickets type I or pseudo-vitamin D deficiency rickets, was effectively treated with physiologic doses of 1,25-dihydroxyvitamin D_3_ [50,51]. Both active vitamin D compounds were also effectively used to treat hypocalcemia in patients with hypoparathyroidism and pseudohypoparathyroidism [52,53]. These clinical successes prompted Dr. Milan Uskokovic at Hoffmann LaRoche to develop a streamlined method for producing the active metabolite, and it became commercially available as an FDA-approved pharmaceutical in the early 1970s.

## 9. Enter the Era of Noncalcemic Genomic Health Benefits of Vitamin D

In 1979, Stumpf et al. reported that ^3^H-1,25(OH)_2_D_3_ was concentrated in the nuclei of most tissues in the body of a vitamin-D-deficient rat [54]. This provided evidence that the vitamin D receptor (VDR) not only existed in calcium-regulating tissues, but also was present in tissues that were not related to calcium and bone metabolism. The physiologic significance of this revelation was not appreciated until it was observed that 1,25(OH)_2_D_3_ inhibited the proliferation and induced terminal differentiation of HL-60 human myeloid leukemia cells [55]. Several laboratories in the early 1980s began to report that some cultured cancer cells possessed a VDR and that incubation of these cells with 1,25(OH)_2_D_3_ resulted in decreased proliferative activity [56]. The observation that mice with M1 leukemia had prolonged survival when they were treated with 1α(OH)D_3_ prompted great interest in seeing whether the same could be true for patients with preleukemia [57]. Eighteen patients with preleukemia were treated with 1,25(OH)_2_D_3_. Although the patients performed quite well early in the treatment, they ultimately developed hypercalcemia, and all died in blastic phase [58]. As a result of these initial studies, a huge effort was made to develop 1,25(OH)_2_D_3_ and its active analogs as a potential therapy for treating a variety of cancers, including prostate cancer. Unfortunately, these therapies were not found to be successful [59]. It appeared that the cancer cells cleverly designed mechanisms to bypass the antiproliferative and pro-differentiating properties of 1,25-dihydroxyvitamin D_3_ and its active analogs. As a result, 40+ years later, there still has never been a vitamin D analog that has proven to be successful in treating any form of cancer.

**Figure 12:**
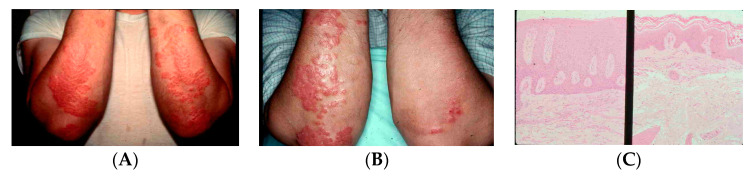
(**A**) A young man with chronic psoriasis with no effective treatment. He was a participant in our clinical research trial evaluating the effectiveness of topical-applied 1,25-dihydroxyvitamin D3 that I formulated in Vaseline with a concentration of 50 mcg/gram. This was a double-blind placebo-controlled trial with one side receiving topical Vaseline and the other side receiving topical Vaseline containing 1,25-dihydroxyvitamin D3. (**B**) After 2 months, there was dramatic improvement on the right forearm that had received the active metabolite. (**C**) A skin biopsy was taken from each forearm and the histology of the skin biopsy from the right forearm shown on the right side of figure (**C**) revealed a dramatic improvement by marked decreases in the keratinocyte proliferation with induction of terminal differentiation to normalize the skin. Holick copyright 2023. Reproduced with permission.

At the same time, Bikle et al. [60] and our group [61] discovered that keratinocytes had a VDR, and when these cells were incubated with 1,25(OH)_2_D_3_, they demonstrated a dramatic response. The keratinocytes decreased their proliferative activity and became fully differentiated. It had been reported that one osteoporotic patient being treated with 1α-hydroxyvitamin D_3_ had improvement in their psoriasis. We had at the same-time initiated psoriasis clinical trials with both topical and oral 1,25(OH)_2_D_3_, which demonstrated remarkable improvement in disease activity (Figure 12). Pharmaceutical companies began making analogs to see if they could reduce the calcemic action of 1,25(OH)_2_D_3_ while maintaining the same or more potent anti-proliferative and pro-differentiating activity. Several analogs were developed, and they and 1,25(OH)_2_D_3_ remain a first-line treatment for patients with minimal psoriasis [62,63,64,65].

**Figure 13:**
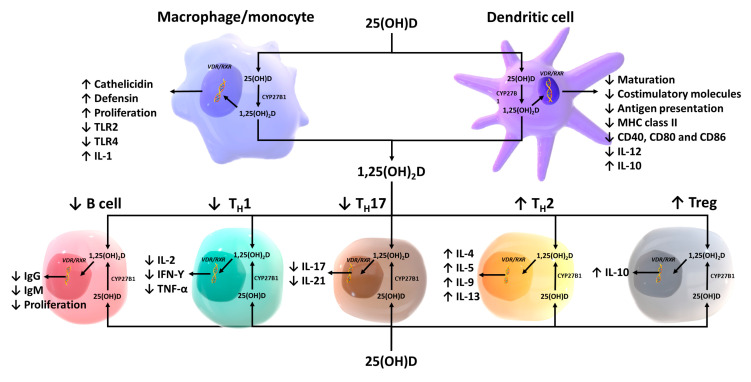
Schematic representation of paracrine and intracrine function of vitamin D and its metabolites and actions of 1,25-dihydroxyvitamin D on the innate and adaptive immune systems. Abbreviations: 1,25(OH)2D: 1,25-dihydroxyvitamin D; 25(OH)D: 25-hydroxyvitamin D, IFN-Y: interferon-Y; IL: interleukin; MHC: membrane histocompatibility complex, TH1: T helper 1; TH2: T helper 2; TH17: T helper 17; Treg: regulatory T cell, TNF-α: tumor necrosis factor-α; TLR2: toll-like receptor 2; TLR4: toll-like receptor 4. Ref. [67] Holick MF, copyright 2020. Reproduced with permission.

**Figure 14:**
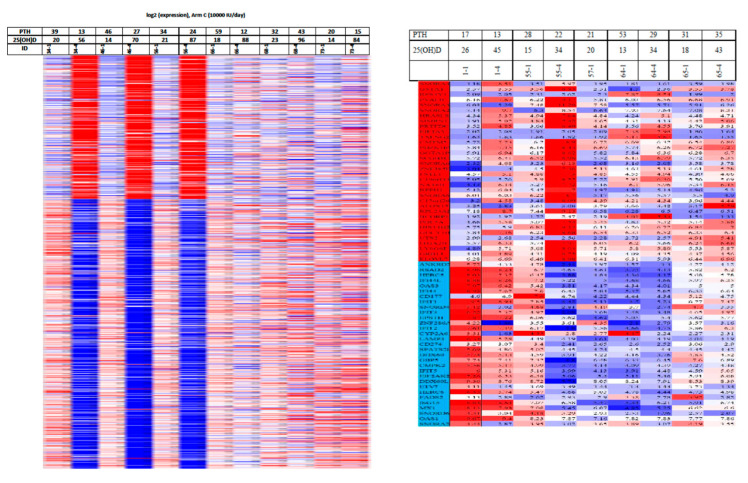
(**Left**) Heatmap of 1289 vitamin D-responsive genes whose expression response variation in 6 vitamin-D-deficient subjects taking 10,000 IUs per day of vitamin D_3_ for 6 months, showing that 3 subjects had a robust response in gene expression compared to the other 3 subjects who had minimum to modest responses even though these subjects raised their blood levels of 25(OH)D in the same range of ~60–90 ng/mL. (**Rgiht**) Heatmap of only 128 vitamin D-responsive genes in 5 vitamin D deficient subjects taking 600 IUs per day for 6 months. Abbreviation: 0 m: 0 month; 6 m: 6 months; 25(OH)D: 25-hydroxyvitamin D; PTH: parathyroid hormone. Ref. [68] Holick MF, copyright 2019. Reproduced with permission.

Although it was dogma that only the kidneys could produce 1,25(OH)_2_D_3_, many laboratories began reporting that a wide variety of tissues and cells not only had a vitamin D receptor but also expressed the 25-hydroxyvitamin D-1α-hydroxylase. Of particular interest was the observation that when macrophages were activated, toll-like receptors were turned on to induce the macrophage to express the 25-hydroxyvitamin D-1α -hydroxylase. This resulted in the ability of the macrophage to metabolize 25(OH)D_3_ to 1,25(OH)_2_D_3_ (Figure 7). The reason for this activation was that once produced, 1,25(OH)_2_D_3_ interacted with the macrophage VDR, resulting in expression and production of cathelicidin, a defensen protein responsible for killing and lysing infectious agents, including bacteria and viruses [66]. It is also now recognized that 1,25(OH)_2_D_3_ produced by immune cells is a major regulator of both innate and acquired immunity, as illustrated in Figure 13 [67]. When healthy vitamin-D-deficient/insufficient adults were given 600 IUs or 10,000 IUs daily for 6 months, an evaluation of their circulating immune cells before and after revealed that even on 600 IUs daily, 128 genes were being influenced, whereas 1289 genes were being influenced in those taking 10,000 IUs daily [68]. What was also observed was although all the healthy vitamin-D-deficient/insufficient participants substantially raised their circulating concentrations of 25(OH)D in the range of 60–90 ng/mL, only 50% had a robust response. This observation demonstrates that there were other factors such as VDR polymorphisms that could influence the responsiveness of vitamin D, promoting genomic effects on the immune system (Figure 14)

## 10. Associating Vitamin D Deficiency with Acute and Chronic Illnesses

Most tissues and cells in the body have a VDR and can produce 1,25(OH_)2_D in a wide variety of cells, including prostate, breast, colon, skin and the brain. The reason that the local production of 1,25(OH)_2_D_3_ does not potentially cause hypercalcemia is that once it is produced and enters the nucleus, 1,25(OH_)2_D immediately induces the 25-hydroxyvitamin D-24-hydroxylase. This enzyme begins to rapidly metabolize 1,25(OH)_2_D_3_ to an inactive water-soluble carboxylic acid derivative (calcitroic acid). Simultaneously, 1,25(OH)_2_D_3_ up- and downregulates more than 1000 genes responsible for cellular proliferation, differentiation, a variety of cellular metabolic activities, antiangiogenesis and apoptosis (Figure 7) [30,68].

**Figure 15:**
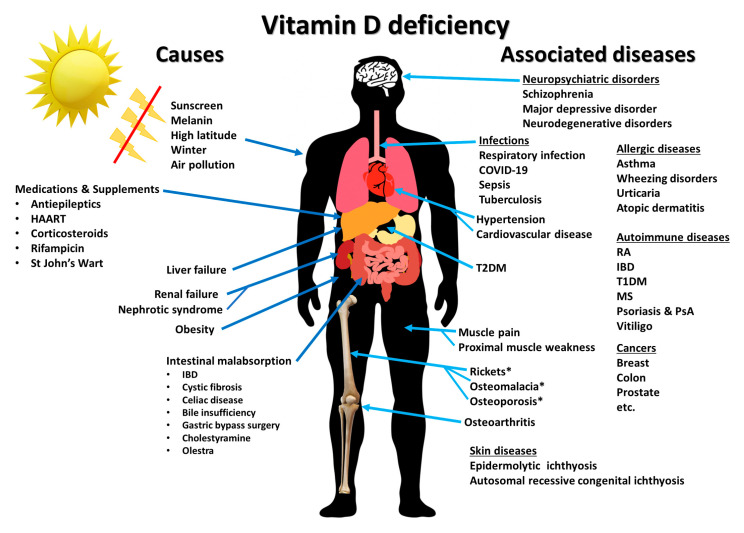
Summary of causes of vitamin D deficiency and diseases and disorders associated with vitamin D deficiency. Abbreviations: HARRT: highly active antiretroviral therapy; IBD: inflammatory bowel diseases; MS: multiple sclerosis; PsA: psoriatic arthritis; T1DM: type 1 diabetes mellitus; T2DM: type 2 diabetes mellitus; RA: rheumatoid arthritis. * denotes diseases that are direct consequences of vitamin D deficiency. Ref. [67] Holick MF, copyright 2020. Reproduced with permission.

Epidemiology studies have related vitamin D deficiency with a multitude of chronic illnesses, including the autoimmune disorders multiple sclerosis, type 1 diabetes and rheumatoid arthritis, cardiovascular disease, type 2 diabetes, neurocognitive dysfunction and infectious diseases, including COVID-19 (Figure 15) [69,70,71]. These observations will be discussed in more detail by other authors participating in this special edition.

## 11. The Future of Vitamin D for the Next 100 Years

The discovery of the antirachitic nutrient/hormone, vitamin D, by McCollum 100 years ago has had and continues to have far-ranging health benefits. This is due to the discovery that vitamin D must undergo sequential metabolism in the liver to 25(OH)D, which is then converted to its active form, 1,25(OH)_2_D, in the kidneys for regulating calcium and phosphate metabolism. The new appreciation that essentially all organs and cells have a VDR, and that many cells can produce 1,25(OH)_2_D, opens a new chapter for vitamin D playing an important role in promoting good health and well-being by reducing the risk for chronic illnesses (Figure 7 and Figure 15). 

Vitamin D has by no means revealed all its biologic functions and clinical potential. It is hoped that there will be a resolution for recommendations for how much vitamin D and sensible sun exposure is necessary for maximum health. There continues to be debate as to what the definition of vitamin D deficiency is based on a circulating blood concentration of 25(OH)D. There is consensus that to achieve and maintain a healthy skeleton, the circulating concentration of 25(OH)D should be at least 20 ng/mL. To maintain maximum bone health and to prevent any evidence of vitamin D deficiency osteomalacia, the circulating concentration should be at least 30 ng/mL [72]. There is mounting evidence that when a blood concentration of 25(OH)D is at least 30 ng/mL, the noncalcemic health benefits of vitamin D become more apparent, based on association studies and randomized controlled trials. It is documented that Maasai herders and the Hazda maintain a circulating concentration of 25(OH)D in the range of 40–60 ng/mL. This concentration range has been associated with many of the noncalcemic health benefits including reduced risk for cardiovascular disease, neurocognitive dysfunction, several cancers and infectious diseases [73]. To achieve a circulating concentration of 25(OH)D in the range of 40–60 ng/mL requires a daily average intake of 2000–5000 IUs or the same amount of vitamin D_3_ produced in the skin from sun exposure, as demonstrated in the Maasai and Hazda. An adult in a bathing suit exposed to enough sunlight to cause a minimal erythemal response results in the production of an amount of vitamin D equivalent to ingesting 15,000–20,000 IUs of vitamin D [72].

There is some evidence to suggest that vitamin D itself has its own health benefits. When screening for compounds that were most effective in stabilizing endothelial membranes, vitamin D_3_ was found to be much more effective than its metabolites as well as a multitude of other compounds [74]. Extremely high doses, in the range of 20,000–50,000 IUs daily, have been effective in treating autoimmune disorders including psoriasis, multiple sclerosis, rheumatoid arthritis and vitiligo. The toxicity that is associated with ingesting these high doses of vitamin D is mitigated by having the patients on an extremely low calcium diet and maintaining good hydration [67,75].

It is likely in the future with the advent of the UVB-emitting LEDs that a variety of devices will become available for personalized in-home use for generating adequate amounts of vitamin D in the skin [76]. At this time, it is generally accepted that obtaining vitamin D from supplements and diet is the same as obtaining vitamin D_3_ from sun exposure. There is evidence that during sun exposure, not only previtamin D_3_ is produced in the skin, but there are other photoproducts of both previtamin D_3_ and vitamin D_3_ that have unique biologic activities not related to the classical nuclear function through the VDR [77] (Figure 7).

The reintroduction of 25(OH)D_3_ (calcifediol) for the treatment of vitamin D deficiency offers the advantage of its hydrophilic properties by being more bioavailable in patients with fat malabsorption syndromes and obesity. In the future, we may see a combination supplement containing 25(OH)D_3_ and vitamin D_3_ [78].

The enthusiasm for the health benefits of vitamin D, which was initiated 100 years ago with its identification by McCollum, has not abated and continues to prosper.
